# Commentary: The Cognitive Costs of Context: The Effects of Concreteness and Immersiveness in Instructional Examples

**DOI:** 10.3389/fpsyg.2016.01608

**Published:** 2016-10-19

**Authors:** Gabriel A. Radvansky

**Affiliations:** Psychology, University of Notre DameNotre Dame, IN, USA

**Keywords:** memory, learning, context, concreteness, ecological validity, training, long-term memory

In their paper, Day et al. (2015) present a pair of studies exploring whether the concreteness of learned material and the context in which learning is embedded can influence retention. This builds on prior work assessing the influence of concrete examples during learning (Goldstone and Sakamoto, 2003). That work found that while concrete examples can facilitate immediate learning (Bransford and Johnson, 1972), it impedes the ability to transfer that knowledge to new situations, as is found in work on problem solving (Gick and Holyoak, 1983). This commentary highlights and extends three issues touched on and implicated by Day et al.'s paper, but which were not the central focus of that work. These are, namely, the importance of ecological validity, the transfer of learning to new domains, and the exploration of very long-term memories.

## Ecological validity

One issue that has dogged laboratory research in psychology is that many of the findings involve data derived from laboratory studies, which are highly constrained and artificial. Questions have been raised about the ecological validly of such work (Neisser, 1976). How well will our efforts transfer to the real world? Ideally, the findings about memory derived from laboratory work should provide a set of principles that can be applied to real world problems to increase the retention of new information by people in the classroom, the workplace, and so on.

The finding of a detrimental effect of concreteness in the laboratory is an interesting and counter-intuitive results. Concerns about ecological validity of such laboratory research, along with other memory findings, such as the finding that it is easier to remember concrete words better than abstract ones (Paivio, 1965), motivated this work. Thus, intuition suggests that student should learn and remember more when information is presented in a concrete form. Furthermore, Day et al. (2015) make a case for why placing students in a more realistic and natural environment could lead to a positive influence of concreteness. And, yet, intuitive expectations were not fulfilled. Day et al. (2015) were able to show that this counter-intuitive finding is not only observed in controlled, artificial laboratory conditions, but also in more free-form, messy classroom conditions. The fact that this effect was observed with different materials and with different sorts of students underscores the robustness of their finding. Thus, this finding further supports the utility of laboratory-based research, along with the verification of findings under more real-world conditions. Establishing the ecological validity of our findings helps solidify our certainty in our theoretical explanations.

## Transfer of cognition

One of the hot topics in cognition and memory research has been a focus on working memory training and whether this can transfer to tasks that are superficially unrelated to the training paradigm. In these studies, people practice a subset of working memory tasks. Afterward there is an assessment of whether this leads to improvements on other tasks, thereby showing a training benefit. While some work has suggested that such transfer is possible (Au et al., 2015), other work takes a more doubtful stance (Redick et al., 2013).

Although not explicitly identified as such, a similar emphasis on the success of transfer occurs in Day et al.'s (2015) paper. However, rather than trying to train up cognition to do a certain type of task, or to modify a basic aspect of cognition, such as working memory, the concern was with the degree to which different forms of knowledge, concrete or abstract, lead to improved performance on superficially different, but conceptually analogous tasks. Thus, the issue was how different kinds of learning examples facilitated or inhibited this transfer. The Day et al. finding was that transfer was more successful with more abstract materials, even though the basic task was the same throughout. Thus, this motivates further work assessing the degree to which prior learning situations can improve the transfer of new knowledge from one domain to another.

## Very long term memory

Day et al.'s (2015) study also looked at the long term consequences of a manipulation on memory, at least for the first experiment. While we have been studying memory for well over a century, the vast majority of studies on this topic do not look at retention for periods longer than an hour. This is, in large part, likely due to the continued influence of the modal model of memory (Atkinson and Shiffrin, 1968) which no one believes and everyone uses. For the typically way of thinking about the modal model, information in short-term memory hangs around for about 30 s if it is not actively rehearsed. If it is rehearsed to a sufficient degree, then it may be transferred to long-term memory. Long-term memory is thus anything that is remembered outside of this 30 s window. Things that are remembered 10 min are treated as being similar to things that are remembered 10 weeks later, although less will be remembered following the forgetting curve.

Of course, you don't need to press memory researchers very hard for them to acknowledge that longer-term memory can be affected by a variety of other process, such as the influence of sleep, interfering material that people encountered during the day, and so on. Yet, still, most do not take these factors into account in their own studies of human long-term memory. More to the point, as a research community we have no idea of the degree to which different factors over time actually influence memory. How much does sleep actually change memory? How interfering is the material encountered during the day? We need more very long-term assessments of memory, as was done by Day et al. (2015), to better understand the processes of retention and forgetting.

## Summary

The research reported by Day et al. (2015) highlights important current and developing issues in the study of learning and memory. Consistent with laboratory studies, they found that testing students in a more naturalistic environment did not reverse a cost observed in using more concrete materials during learning. Although directly emphasized in their paper, this work highlights the value in extending laboratory work more ecologically valid circumstances, the ability of different kinds of training experiences to transfer to novel circumstances, and the very long-term retention of knowledge.

## Author contributions

The author is solely responsible for crafting this commentary.

### Conflict of interest statement

The author declares that the research was conducted in the absence of any commercial or financial relationships that could be construed as a potential conflict of interest.
